# Comparative Effectiveness of Botulinum Toxin Injection for Chronic Shoulder Pain: A Meta-Analysis of Randomized Controlled Trials

**DOI:** 10.3390/toxins12040251

**Published:** 2020-04-12

**Authors:** Po-Cheng Hsu, Wei-Ting Wu, Der-Sheng Han, Ke-Vin Chang

**Affiliations:** 1 Department of Physical Medicine and Rehabilitation, National Taiwan University Hospital, Bei-Hu Branch, Taipei 10845, Taiwan; myronrbman@gmail.com (P.-C.H.); wwtaustin@yahoo.com.tw (W.-T.W.); dshan1121@yahoo.com (D.-S.H.); 2 Institute of Clinical Medicine, National Yang-Ming University, Taipei 11221, Taiwan; 3 Department of Physical Medicine and Rehabilitation, National Taiwan University College of Medicine, Taipei 10048, Taiwan

**Keywords:** botulinum toxin, corticosteroid, joint, myofascial pain

## Abstract

Botulinum toxin (BoNT) injection is regarded as a promising treatment for musculoskeletal pain. However, its efficacy for treating chronic shoulder pain remains unclear. We investigated the effectiveness of BoNT injections for chronic shoulder pain by conducting a systematic search of electronic databases up to March 2020 for randomized control trials (RCTs) that used BoNT injections for chronic shoulder pain treatment. The primary outcome was the between-group comparison of pain reduction, quantified by the standardized mean difference (SMD). Nine RCTs comprising 666 patients were included and divided into two groups: one group with shoulder joint pain (*n* = 182) and the other group with shoulder myofascial pain (*n* = 484). Regarding shoulder joint pain, the efficacy of BoNT injections was similar to that of the reference treatment (SMD: −0.605, 95% confidence level [CI]: −1.242 to 0.032 versus saline; SMD: −0.180, 95% CI: −0.514 to 0.153 versus corticosteroids) at one month post-intervention, and was superior (SMD: −0.648, 95% CI: −0.1071 to −0.225 versus corticosteroids) between one and three months. Likewise, in terms of shoulder myofascial pain, the effectiveness of BoNT injections did not differ from the reference treatment (SMD: −0.212, 95% CI: −0.551 to 0.127 versus saline; SMD: 0.665, 95% CI: −0.260 to 1.590 versus dry needling and SMD: 1.093; 95% CI: 0.128 to 2.058 versus lidocaine) at one month post- intervention, and appeared superior (SMD: −0.314, 95% CI: −0.516 to −0.111 versus saline) between one and three months. Our meta-analysis revealed that BoNT injections could be a safe and effective alternative for patients with chronic shoulder pain.

## 1. Introduction

Shoulder pain, a prevalent musculoskeletal disorder, is estimated to affect 16% of the general population [1]. The lifetime prevalence of shoulder pain could be up to 67%, according to a systemic review [2]. Shoulder pain not only decreases work efficiency and life quality, but also leads to an increased economic and medical burden on the whole society [1].

The shoulder is a complicated structure. Rotator cuff tendon disorders and related bursal and joint pathology are the most common causes of shoulder pain. However, myofascial pain, characterized by the presence of myofascial trigger points (MTrPs), can develop over the shoulder region. Its diagnostic criteria consist of taut bands, hypersensitive spots, and referred pain over the affected area, and MTrPs are usually treated as the therapeutic targets [3].

Injection is an effective treatment for musculoskeletal disorders, and the commonly used injectates include corticosteroids, local anesthetics, hyaluronic acid, and high-concentration dextrose [4,5,6]. Botulinum toxin (BoNT) has been widely used in managing post-stroke spasticity, hemifacial spasm, and cervical dystonia. An increasing amount of evidence suggests that BoNT not only inhibits acetylcholine release at the neuromuscular junction, but also modulates pain by reducing nociceptive neurotransmitters and neurogenic inflammation [7]. In recent years, BoNT injections have been increasingly used to treat mild musculoskeletal disorders, like tennis elbow, knee osteoarthritis, and refractory joint pain [8,9,10]. However, its effects remain inconclusive regarding chronic shoulder pain based on available systemic reviews and meta-analyses [11,12]. Wu et al. concluded that BoNT injections were effective in treating shoulder pain compared with placebo injections [12]. However, their meta-analysis included stroke patients with hemiplegic shoulders, so the results might not be well-representative of the general population. Conversely, Ahmed et al. demonstrated that the local anesthetic injections were more effective than BoNT injections [11]. Nevertheless, their patients had heterogeneous symptoms and some of them presented with myofascial pain over all body regions. In this regard, this meta-analysis aimed to clarify whether BoNT injections were effective for the treatment of chronic shoulder pain in the general population through reviewing evidence from randomized controlled trials (RCTs).

## 2. Results

### 2.1. Study Identification and Selection

**Figure 1:**
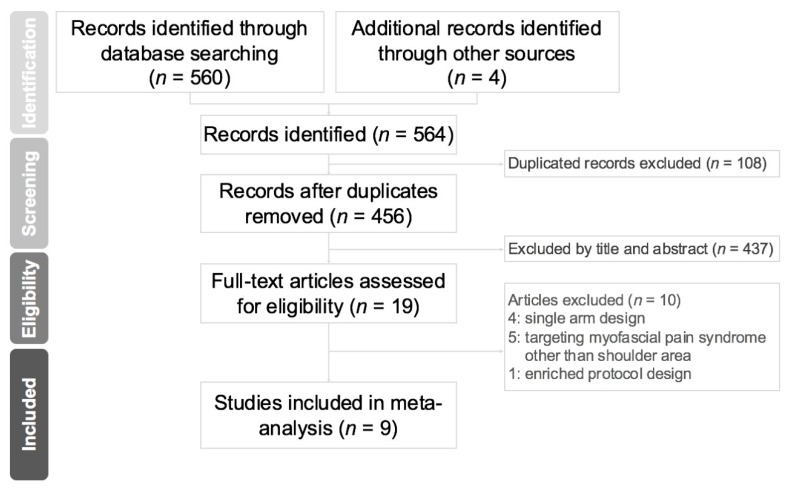
Preferred Reporting Items for Systematic Reviews and Meta-Analyses (PRISMA) flow diagram for the study selection process.

The initial search of the databases yielded 564 studies. After excluding 108 duplicated articles and 439 non-relevant articles by screening titles and abstracts, 19 studies were deemed eligible for subsequent evaluation. Ten were further excluded because four lacked a control group [10,13,14,15], five targeted myofascial pain in regions other than shoulder areas [16,17,18,19,20], and one used an enriched protocol design to assess the effect of repeated BoNT injections [14] (Figure 1).

The final meta-analysis consisted of seven 2-armed [21,22,23,24,25,26,27], one 3-armed [28], and one 4-armed [29] trials. Of the four studies investigating shoulder joint pain, one targeted patients with refractory shoulder pain [27], one targeted those with subacromial bursitis or shoulder impingement syndrome [25], one targeted those with adhesive capsulitis [24], and one was shoulder osteoarthritis [23]. The other five studies focused on patients with myofascial pain syndrome over the shoulder region [21,22,26,28,29]. This final meta-analysis included 666 participants, 62.5% of whom were women.

**Figure 2:**
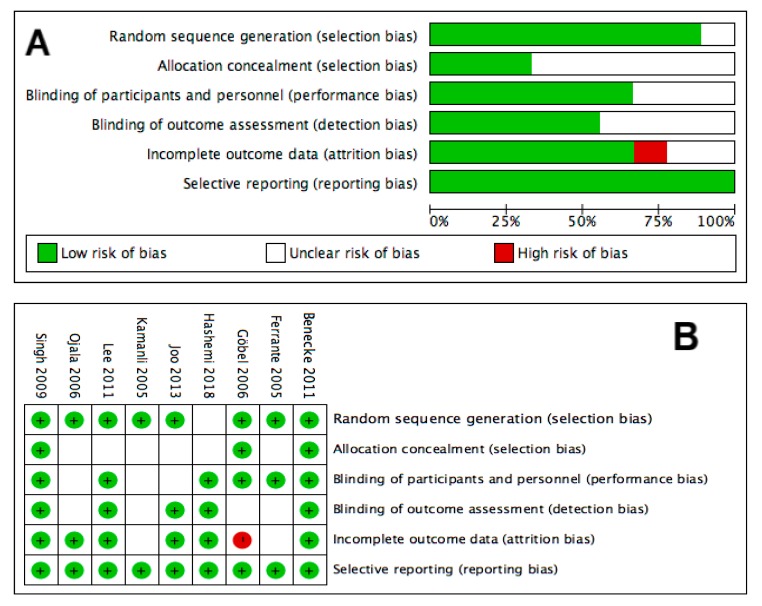
Summary graph (**A**) and table (**B**) of risk for bias of enrolled studies. Green (+): low risk of bias; red (-): high risk of bias; blank: unclear risk of bias.

Regarding the reference treatments in the four studies targeting shoulder joint pain, one employed 0.9% saline injections [27] and three employed corticosteroid injections [23,24,25]. In terms of the five studies for shoulder myofascial pain, four used 0.9% saline [21,22,26,29] and one employed lidocaine and dry needling in two different arms [28]. The details of included studies are documented in Table 1, and the quality assessments are shown in Figure 2.

The type of BoNT used in the included studies was predominantly BoNT-A; only one study used BoNT-B (Myobloc^®^, Solstice Neurosciences, Inc., South San Francisco, CA, USA) [25]. Among the studies using BoNT-A, two used Botox^®^ (Allergan, Irvine, CA, USA) [26,27], two used Dysport^®^ (Ipsen Ltd., Ipsen Biopharm Ltd., Wrexham, UK) [21,22,24], and three studies did not specify the injections [23,28,29]. The dosage of BoNT varied across different brands. Regarding shoulder joint pain, the common dosage for injection was 100 U of Botox^®^ (BoNT-A), 200 U of Dysport^®^ (BoNT-A) or 2500 U of Myobloc^®^ (BoNT-B). In terms of shoulder myofascial pain, the dosage for injection per trigger point ranged from 5 to 40 U of BoNT-A. The details of regimens, injection techniques, outcome measurements, and follow-up durations are listed in Table 2.

### 2.2. Outcomes

#### 2.2.1. Shoulder Joint Pain

**Figure 3:**
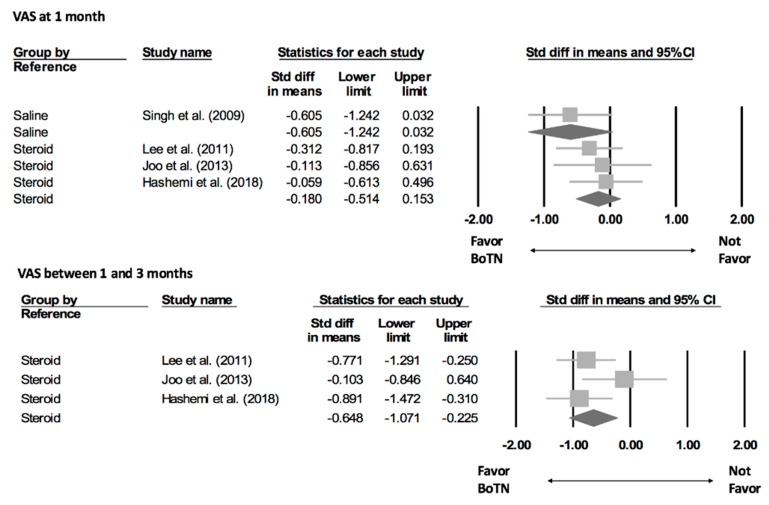
Forest plot of pain reduction from botulinum toxin injection for shoulder joint pain at 1 month and between 1–3 months after injection. VAS, visual analogue scale of pain. The square indicates the point estimate and the rhombus represents the pooled effect size.

Regarding the visual analogue scale (VAS) at one month after intervention, only one trial compared BoNT injections with saline [27]; the standardized mean difference (SMD) was −0.605 (95% CI, −1.242 to 0.032) (Figure 3). The other three trials used corticosteroid injections as comparisons and their summarized SMD was −0.180 (95% CI, −0.514 to 0.153; I square < 0.001) [23,24,25]. None of the aforementioned effect sizes reached statistical significance. In terms of VAS between one and three months after intervention, only the studies comparing corticosteroid injections had available data. The pooled SMD was −0.648 (95% CI, −0.1071 to −0.225), indicating a significantly superior outcome of BoNT than corticosteroids.

#### 2.2.2. Shoulder Myofascial Pain

**Figure 4:**
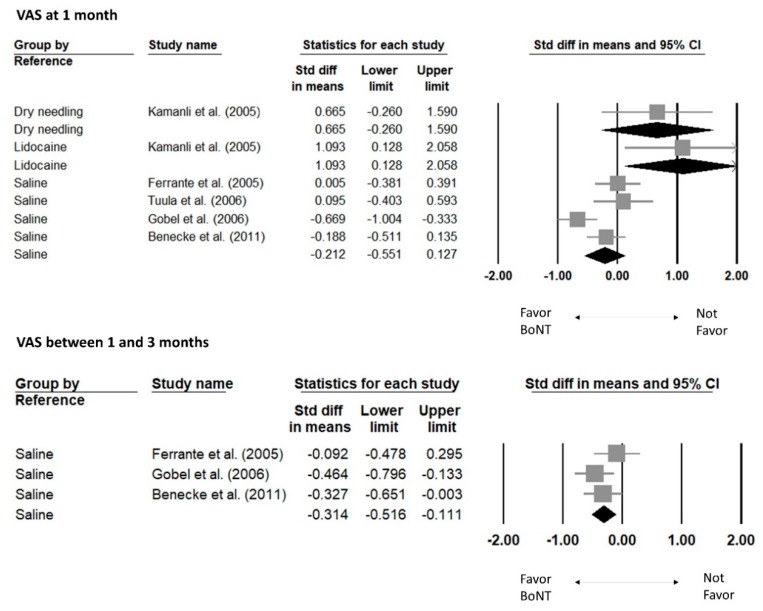
Forest plot of standardized mean differences in pain reduction of botulinum toxin versus reference treatment for myofascial pain over the shoulder region within 1 month, and 1–3 months after injection. The square indicates the point estimate and the rhombus represents the pooled effect size.

In a triple-arm trial, the SMDs of the VAS at one month post- intervention were 0.665 (95% CI, −0.260 to 1.590) comparing dry needling, and 1.093 (95% CI, 0.128 to 2.058) comparing lidocaine injections [28] (Figure 4). A 4-armed RCT with 3 different doses of BoNT compared with saline was merged into one group to compare the effects after saline injections [29]. Four studies compared saline injections, and the pooled effect size at one month post- intervention was −0.212 (95% CI, −0.551 to 0.127; I square: 68.8%) [21,22,26,29]. Regarding the VAS between one and three months, the summarized SMD comparing saline from 3 studies was −0.314 (95% CI, −0.516 to −0.111; I square: 3.3%), showing a significant effect favoring BoNT injections.

### 2.3. Adverse Events

Among the 9 enrolled studies, 8 trials clearly reported the adverse events [21,22,24,25,26,27,28,29]. Most documented adverse events related to BoNT injections were of mild to moderate severity, such as temporary muscle pain or soreness after treatments. In one study [27], three cases in the BoNT-A group and nine cases in the reference group reported serious adverse events after injections (Table 2). However, most of the events (e.g., chest pain, atrial fibrillation, abdominal pain) were related to their underlying diseases and not to the BoNT injections.

### 2.4. Publication Bias

#### 2.4.1. Shoulder Joint Pain

Significant publication bias was detected using the Egger test (*p* = 0.03) regarding the VAS at one month post-injection for myofascial pain. However, there was no significant publication bias between one to three months post-injection. The corresponding funnel plot is shown in Figure S1.

#### 2.4.2. Shoulder Myofascial Pain

The *p*-value of the Egger test for the VAS at one month was 0.047, indicating significant publication bias. No significant publication bias was detected regarding the VAS between one to three months post-injection. The corresponding funnel plot is shown in Figure S2.

## 3. Discussion

### 3.1. Summary of the Meta-Analysis

The present meta-analysis included available RCTs to investigate the efficacy of BoNT injections in chronic shoulder pain, revealing that the BoNT injection was similar to the reference treatments regarding short-term effectiveness. Compared with corticosteroid and saline injections, BoNT injections yielded more symptom relief between one to three months following treatment in terms of joint and myofascial pain over the shoulder region.

### 3.2. Botulinum Toxin (BoNT) Injection in Shoulder Joint Pain

Pain over the shoulder joint is predominantly derived from degeneration and chronic inflammation of the rotator cuff tendons and glenohumeral joint. Corticosteroid injections are widely used in the management of shoulder pain based on its anti-inflammatory potential, and its effectiveness has been proven by several clinical studies [30,31,32,33]. On the other hand, animal studies demonstrated that BoNT injections could inhibit release of pain mediators, including glutamate, substance P, and calcitonin gene-related peptides [34,35,36]. Hence, based on our analysis, inflammatory shoulder pain might be modulated by blocking nociception by injecting BoNT, whose short-term efficacy has been shown to be similar to corticosteroid injections.

However, a significantly better outcome of BoNT injections was observed at the mid-term follow-up, implying a more durable effect of BoNT than corticosteroids. Recurrent pain after corticosteroid injections was common [37,38], although the timing of the recurrence varies with initial clinical manifestation and disease severity [32]. Possible explanations for the lasting effect of pain reduction after BoNT injections include: (1) the duration of action of BoNT was longer than corticosteroids (approximately 3 months vs. 8 weeks) [37,39] and (2) BoNT inhibits not only pain mediators, but also impedes downstream neurogenic inflammation [40,41]. Neurogenic inflammation is a phenomenon of reduced sensory nerve thresholds and increased activation of sensory neurons, induced by excitation of nociceptors following the stimulation of pain mediators [42]. Therefore, pain relief after BoNT injections in chronic shoulder joint pain might be attributed to the interference of afferent pain signals and modulation of neurogenic inflammation, which could not be achieved by the administration of corticosteroids [43].

### 3.3. BoNT Injection in Shoulder Myofascial Pain

For shoulder myofascial pain, our meta-analysis revealed no significant difference in short-term efficacy between BoNT injections and other comparative treatments, like dry needling and injection of saline and lidocaine. Immediate analgesia after injections on MTrPs can be attributed to the needling effect or temporary analgesia after local anesthetics [44]. Moreover, an injection itself may increase muscle circulation [45], which could be achieved both in the BoNT injections and reference groups. Nevertheless, our results revealed that BoNT injections had better mid-term pain relief than saline injections, possibly resulting from the pharmacological effect of BoNT. Some studies showed that pain from MTrPs was the consequence of dysfunction at the motor endplates with subsequent chronic muscle contraction [46]. Administration of BoNT on MTrPs has been shown to reduce acetylcholine release at the neuromuscular junction possible relief of myofascial pain after muscle relaxation. Moreover, the accumulation of oxidative stress and the depletion of the energy supply in MTrPs can reinforce peripheral sensitization of nociception, leading to centralization of pain perception [47]. BoNT injections may inhibit pain mediators released peripherally to decrease central sensitization. Importantly, the average duration of action of BoNT is 12 weeks [48], which also explained why BoNT injections had a longer effect on shoulder myofascial pain than saline injections.

### 3.4. Type and Dosage of BoNT for Injection

Among all the enrolled studies, only one trial used BoNT-B (Myobloc^®^) [25]. This was probably due to higher post-injection pain, more symptoms of dysautonomia, shorter duration of action, and an increased incidence of immunogenicity after the administration of BoNT-B. [49,50]. According to previous literature [51,52], the effective dosage of 100 units (U) of Botox^®^ is equivalent to 200–300 U of Dysport^®^ or 5000 U of Myobloc^®^. After conversion, we found the dosage of BoNT for intra-bursal injections was roughly half of the dosage for intra-articular injections of our included trials.

Regarding shoulder myofascial pain, the total dosage depends on the dose at each injection site and the number of trigger points. The maximum dose allowed for injections on a patient were predesignated in 3 studies [21,22,26], which was up to 35 U of Botox^®^ [26] and 400 U of Dysport^®^ [21,22]. Caution should be taken regarding the upper limit of BoNT administered per patient, especially for those with preexisting neuromuscular diseases.

### 3.5. Clinical Implications

This meta-analysis is in favor of BoNT injections for the treatment of chronic shoulder pain. Regarding shoulder joint pain, intra-articular or intra-bursal administration of BoNT has a similar short-term, but better mid-term analgesic effect, compared with corticosteroid injections. In terms of shoulder myofascial pain, BoNT injections could achieve longer lasting pain relief than saline injections.

### 3.6. Limitations

This meta-analysis has limitations. First, the patients in the enrolled studies had different etiologies. Therefore, we separated them into two groups for analysis based on their causes of pain. Second, the available follow-up durations of all included trials were only up to three months, so the long-term effects of BoNT injections for chronic shoulder pain is unknown. Third, whether BoNT injections could improve shoulder function was not within the scope of this meta-analysis; future studies are needed to explore these questions. Lastly, the number of the studies eligible for inclusion was relatively small when limiting the research type only in RCTs. This might mitigate the power of the conclusion derived from the present meta-analysis. Nevertheless, some case series investigating BoNT injections also demonstrated pain reduction and functional improvement in shoulder joint and myofascial pain [13,53], which were compatible with our findings.

## 4. Conclusions

BoNT injections had similar short-term efficacy as the reference treatments like corticosteroid and saline injections for relieving chronic shoulder pain. Its effect was superior to the reference treatments between one to three months following injections. The majority of adverse effects pertinent to BoNT injections were temporary and reversible and their severity was mild to moderate. Intra-articular, -bursal, and -muscular administration of BoNT is considered to be a safe and effective alternative for patients with chronic painful shoulders.

## 5. Methods

### 5.1. Search Strategy and Criteria

Two electronic databases, PubMed and Embase, were scrutinized for relevant articles published from the earliest record to Jan 2020. We also searched the Cochrane Central Register of Controlled Trials, Cochrane Database of Systematic Reviews, and ClinicalTrials.gov database for suitable references. Moreover, we manually examined the reference lists of the included articles for pertinent trials. The key terms, “botulinum toxin”, “shoulder”, and “myofascial pain” were entered as the medical subject heading and text words for literature searches. The search strategy is presented in Appendix A. The current meta-analysis was not conducted based on any registered or published protocols.

### 5.2. Inclusion and Exclusion Criteria

The inclusion criteria were as follows: (1) RCTs, (2) enrollment of patients with shoulder pain, including tendon and joint related disorders (including adhesive capsulitis, subacromial impingement and osteoarthritis) and myofascial pain syndrome [54], and (3) quantitative measurements of pain before and after treatments. Furthermore, studies investigating myofascial pain, but not covering the shoulder region, and those recruiting stroke patients with hemiplegic shoulders were not included. Animal studies, case reports, case series, single-arm longitudinal follow-up studies, and quasi-experimental comparative studies were excluded from the present meta-analysis.

### 5.3. Data Collection and Abstraction

Two authors (P.-C.H. and W.-T.W.) independently screened and evaluated whether the retrieved articles were eligible and met the criteria of inclusion. The information of patient demographics (age, gender ratio, disease duration and type, regimen and site for injection, and the parameters of outcome measurements) were recorded by both authors concurrently using the predesignated evaluation form. The corresponding authors would confirm the correctness of the retrieved data.

### 5.4. Assessment of Study Quality

The methodological quality of the enrolled studies was assessed using the Cochrane Risk of Bias Tool for RCTs [55]. The risk of bias was classified as either high, low, or unclear. The quality assessment was based on the following aspects: sequence generation (selection bias), allocation concealment (selection bias), blinding of patients and personnel (performance bias), blinding of outcome assessment (detection bias), incomplete outcome data (attrition bias), and selective outcome reporting (reporting bias). Any discrepancy in opinions of assessments between the two evaluators were solved either through discussion or by the judgment of the corresponding author.

### 5.5. Meta-Analysis Methodology

The change in the VAS of pain before and after treatment was treated as the primary outcome. The VAS was extracted at or closest to the following points: baseline, within one month and between one to three months after the intervention. The summarized SMD of the VAS changes between two treatment arms was used to compare the effectiveness of BoNT injections with other injectates or approaches [56].

The random effects model was applied for pooling of the effect sizes due to the variations in study designs (such as drug dosage, injection technique, and injection numbers) across the included trials. An SMD of 0.2, 0.5, and 0.8 is considered a small, moderate, and large effect size, respectively [57]. The I-square and Cochran’s Q statistics were used to evaluate the degree of heterogeneity among studies. An I-square value of 25%, 50%, and 75% was considered low, moderate, and high heterogeneity, respectively [58]. The symmetry of the effect size distribution on the funnel plot and the result from the Egger’s test were used to assess the potential publication bias. All the analyses were conducted using Comprehensive Meta-analysis Software version 3 (Biostat, Englewood, NJ, USA), and *p* < 0.05 was considered statistically significant.

## Figures and Tables

**Table 1:** Summary of the retrieved trials investigating botulinum toxin on patients with chronic shoulder pain.

Study	Diagnosis	Enrolled Sample Number (Male/Female)	Average Age, Years	Pain Duration, Months	Double Blind	Randomization	Allocation Concealment	Funding Source
**Shoulder Joint Pain**								
Singh et al. 2009 [27]	Refractory shoulder pain	BoNT-A: 21(20/1) Saline: 22 (22/0)	BoNT-A: 72.1 ± 1.9 Saline: 70.2 ± 2.6	BoNT-A:96 ± 24 Saline: 132 ± 36	Yes	Random table	Yes	Arthritis Foundation North Central Chapter grant; VA Scholar grant from the Center for Epidemiological and Clinical Research; Minneapolis VA Medical Center. NIH CTSA Award; Allergan Pharmaceuticals, Inc.
Lee et al. 2011 [25]	Subacromial bursitis or shoulder impingement syndrome	BoNT-B: 31 (14/17) Steroid: 30 (11/19)	BoNT-B: 57.9 ± 10.1 Steroid: 55.8 ± 9.1	BoNT-B:8.2 ± 5.5 Steroid: 8.2 ± 7	Yes	Unclear	Yes	Wooridul Spine Foundation, Korea
Joo et al. 2013 [24]	Adhesive capsulitis	BoNT-A: 15(9/6) Steroid: 13 (8/5)	BoNT-A: 55.0 ± 9.7 Steroid: 53.3 ± 13.7	BoNT-A: 8.7 ± 7.2 Steroid: 5.7 ± 1.5	No	Random table	Unclear	Not mentioned
Hashemi et al. 2018 [23]	Shoulder osteoarthritis	50 (24/26) in total: BoNT-A: 25 Steroid: 25	Mean age: 56 ± 7.6 in total	>3 in total	Yes	Unclear	Unclear	Not mentioned
**Shoulder Myofascial Pain Syndrome**								
Kamanli et al. 2005 [28]	Trigger point located at cervical, back, or shoulder muscles	29 participants (6/23) in total: BoNT-A:9 Lidocaine:10 Dry needling:10	BoNT-A: 38.3 ± 5.26 Lidocaine: 37.30 ± 9.76 Dry needling: 37.20 ± 8.08	BoNT-A: 49.20 ± 34.96 Lidocaine: 32.50 ± 21.99 Dry needling: 50.66 ± 19.92	No	Unclear	Unclear	Not mentioned
Ferrante et al. 2005 [29]	Cervicothoracic and shoulder myofascial Pain	BoNT-A 10U/TP:32 (13/19) BoNT-A 25U/TP:34 (13/21) BoNT-A 50U/TP:31 (11/20) saline: 35 (15/20)	BoNT-A 10U/TP: 43.3 ± 10.9 BoNT-A 25U/TP: 46.6 ± 15 BoNT-A 50U/TP: 46.5 ± 12.21 Saline: 45.3 ± 10.1	>3	Yes	Random table	Unclear	Not mentioned
Ojala et al. 2006 [26]	Neck-shoulder myofascial pain	31 (3/28) in total: BoNT-A: 15 Saline: 16	BoNT-A: 44.9 ± 7.6 Saline: 43.8 ± 8.1	BoNT-A: 10.5 ± 9.9 Saline: 9.5 ± 7.6	Yes	Block randomization	Unclear	Kuopio University Hospital, Finland
Göbel et al. 2006 [22]	Upper back and/or shoulder myofascial pain syndrome	BoNT-A: 74 (16/61) Saline: 70 (16/54)	BoNT-A: 44 ± 12 Saline: 45 ± 11	BoNT-A: 18 ± 6 Saline: 19 ± 9	Yes	Block randomization	Yes	Not mentioned
Benecke et al. 2011 [21]	Myofascial pain syndrome affecting cervical muscles of the back and shoulder	BoNT-A: 76(32/44) Saline: 72 (20/52)	BoNT-A: 48 ± 13 Saline: 45 ± 10	BoNT-A: 19 ± 70 Saline: 19 ± 68	Yes	Block randomization	Yes	Ipsen, UK

BoNT: botulinum toxin.

**Table 2:** Summary of intervention details of botulinum toxin injection in the retrieved trials.

Author, Year	Botulinum Toxin Type (Brand)	Dose/Volume ^*^	Reference Treatment	Injection Technique	Outcome Measurement	Follow Up	Adverse Effects
**Shoulder Joint Pain**							
Singh et al. 2009 [27]	BoNT-A (Botox^®^),	100 U/1 mL pretreated with 2 mL of 1% lidocaine	1 mL of 0.9% saline solution + 2 mL of 1% lidocaine	Landmark guided, posterior approach to glenohumeral joint	VAS; SPADI; ROM (flexion + abduction), short-form McGill Pain Questionnaire; SF-36; proportion of responders	1 month	BoTN-A: 50 AEs (3 serious AEs:2 chest pain,1 scheduled cataract surgery) Placebo: 46 AEs (9 serious AEs:1 chest pain, 2 atrial fibrillation, 1 small-bowel obstruction, 1 abdominal pain, 1 hematuria, 1 acute renal failure, 1 ear pain and 1 anxiety attack)
Lee et al. 2011 [25]	BoNT-B (Myobloc^®^)	2500 U/0.5 mL + 0.5% lidocaine 2 mL	Triamcinolone 40 mg + 2 mL of 0.5% lidocaine	Ultrasound-guided subacromial bursa injection	NRS, DASH, Shoulder ROM (abduction)	1 and 3 months	BoTN-B: 2 AEs (injection site discomfort) Triamcinolone: 3 AEs (injection site discomfort) No serious AEs
Joo et al. 2013 [24]	BoNT-A (Dysport^®^)	200 U/2 mL	Triamcinolone 20 mg (1 mL) + 1 mL of 0.9% saline solution.	Fluoroscopic guidance, anterior approach to glenohumeral joint	NRS; ROM (active flexion, abduction and passive flexion, abduction, external rotation and internal rotation)	2,4 and 8 weeks	BoTN-B: 1 AEs (flu-like symptoms) Triamcinolone: 2 AEs (flu-like symptoms) No serious AEs
Hashemi et al. 2018 [23]	BoNT-A (not mentioned)	100 U/5 mL	Triamcinolone 10 mg (4 mL) + 4 mL of 0.9% saline solution.	Ultrasound-guided glenohumeral joint injection	VAS; ROM (External rotation, internal rotation and abduction)	2 and 12 weeks	Unclear
**Shoulder Myofascial Pain Syndrome**							
Kamanli et al. 2005 [28]	BoNT-A (not mentioned)	10–20 U/1–2 mL (22 injections in 9 patients)	(1) Lidocaine group: 1 mL of 0.5% lidocaine solution (32 injections in 10 patients) (2) Dry needling: empty syringe (33 injections in 10 patients)	Palpation guided trigger point injection	VAS; cervical ROM; Pressure pain threshold; Pain score^§^; Hamilton Anxiety and Depression Inventory; Nottingham Health Profile	1 month	BoNT-A: 9AEs (5 fatigue, 3 muscle pain, 1 headache) Lidocaine: 6 AEs (3 coldness and burning sensation, 3 paresthesia) No serious AEs
Ferrante et al. 2005 [29]	BoNT-A (not mentioned)	10 U/0.5 mL, 25 U/0.5 mL and 50 U/0.5 mL depending on different arms; maximum 250 U on one patient	0.5 mL 0.9% saline	Palpation guided trigger point injection	VAS and sum of pain intensity differences; Rescue medication; Pain pressure threshold; SF-36	0,1,2,3,4,5,6,7,8,12 weeks	BoNT-A: 3 AEs (flu-like symptoms) No serious AEs
Ojala et al. 2006 [26]	BoNT-A (Botox^®^)	5U/0.05 mL (range 15–35 U, mean 28 ± 6 U)	0.05 mL 0.9% saline	Palpation guided trigger point injection	VAS; Self-assessment of the efficacy; Pressure pain threshold	4 weeks	BoNT-A: 7 AEs (1 injection site pain, 2 vertigo, 1 sweating, 1 hands fatigue, 2 headache) Saline: 4 AEs (1 injection site pain, 1 vertigo, 1 hands fatigue, 1 eyelids swelling) No serious AEs
Göbel et al. 2006 [22]	BoNT-A (Dysport^®^)	40 U/0.4 mL (10 trigger points)	0.4 mL of 0.9% saline solution	Palpation guided	Pain intensity^§^	4,8 and 12 weeks	BoNT-A: 31 AEs Saline: 11 AEs No serious AEs
Benecke et al. 2011 [21]	BoNT-A (Dysport^®^)	40 U/0.4 mL (10 fixed injection sites)	0.4 mL of 0.9% saline solution	10 standardized predetermined injection sites in the head, neck, and shoulder.	Pain intensity^§^ Global evaluation of treatment	4,8 and 12 weeks	BoNT-A: 33 AEs Saline: 29 AEs No serious AEs

* The information is shown as the dose and volume per site regarding for the treatment of myofascial pain. ^§^ Pain intensity and Pain score were four-point scale, while 1 (no pain) to 4 (severe pain) for Pain intensity and 0 (no pain) to 3 (severe pain) for Pain score. Abbreviation: BoNT, Botulinum toxin; VAS, Visual Analogue Scale; NRS, Numeric rating scale; SPADI, Shoulder Pain and Disability Index; ROM, range of motion; SF-36, Short form-36; AE, Adverse event.

## Supplementary Materials

The following are available online at https://www.mdpi.com/2072-6651/12/4/251/s1, Figure S1: Funnel plot for the comparisons of the standardized mean difference at (**A**) one month and (**B**) between one and three months after injection for shoulder joint pain, Figure S2: Funnel plot for the comparisons of the standardized mean difference at (**A**) one month and (**B**) between one and three months after injection for shoulder myofascial pain.

## Appendix A. Strategy of Literature Search

**PubMed**

1. (((((((botulinum toxin) OR botox) OR onabotulinumtoxina) OR rimabotulinumtoxinb) OR abobotulinumtoxina) OR incobotulinumtoxin) OR dysport) OR xeomin
2. ((((((shoulder pain) OR rotator cuff) OR bursitis) OR impingement syndrome) OR myofascial pain) OR adhesive capsulitis) OR trigger point
3. (((((randomized controlled trial) OR controlled clinical trial) OR randomized) OR placebo) OR randomly) OR controlled
4. 1 and 2 and 3

**Embase**

1. ‘botulinum toxin’ OR ‘botox’ OR ‘onabotulinumtoxina’ OR rimabotulinumtoxinb OR abobotulinumtoxina OR incobotulinumtoxin OR dysport OR xeomin
2. ‘shoulder pain’ OR ‘rotator cuff’ OR bursitis OR ‘impingement syndrome’ OR ‘myofascial pain’ OR ‘adhesive capsulitis’ OR ‘trigger point’
3. ‘randomized controlled trial’ OR ‘controlled clinical trial’ OR randomized OR ‘placebo’ OR ‘randomly’ OR ‘controlled’
4. 1 and 2 and 3
