# Supplementary material for: Comparative Effectiveness of Botulinum Toxin Injection for Chronic Shoulder Pain: A Meta-Analysis of Randomized Controlled Trials
Source: Toxins (Basel). 2020 Apr 12;12(4):251. doi: 10.3390/toxins12040251 (PMC7232231; doi:10.3390/toxins12040251)
Supplement: Supplementary file 1 [file toxins-12-00251-s001.pdf]

Supplementary Materials: Comparative Effectiveness of Botulinum Toxin Injection for Chronic Shoulder Pain: A Meta-Analysis of Randomized Controlled Trials

Po-Cheng Hsu, Wei-Ting Wu, Der-Sheng Han and Ke-Vin Chang

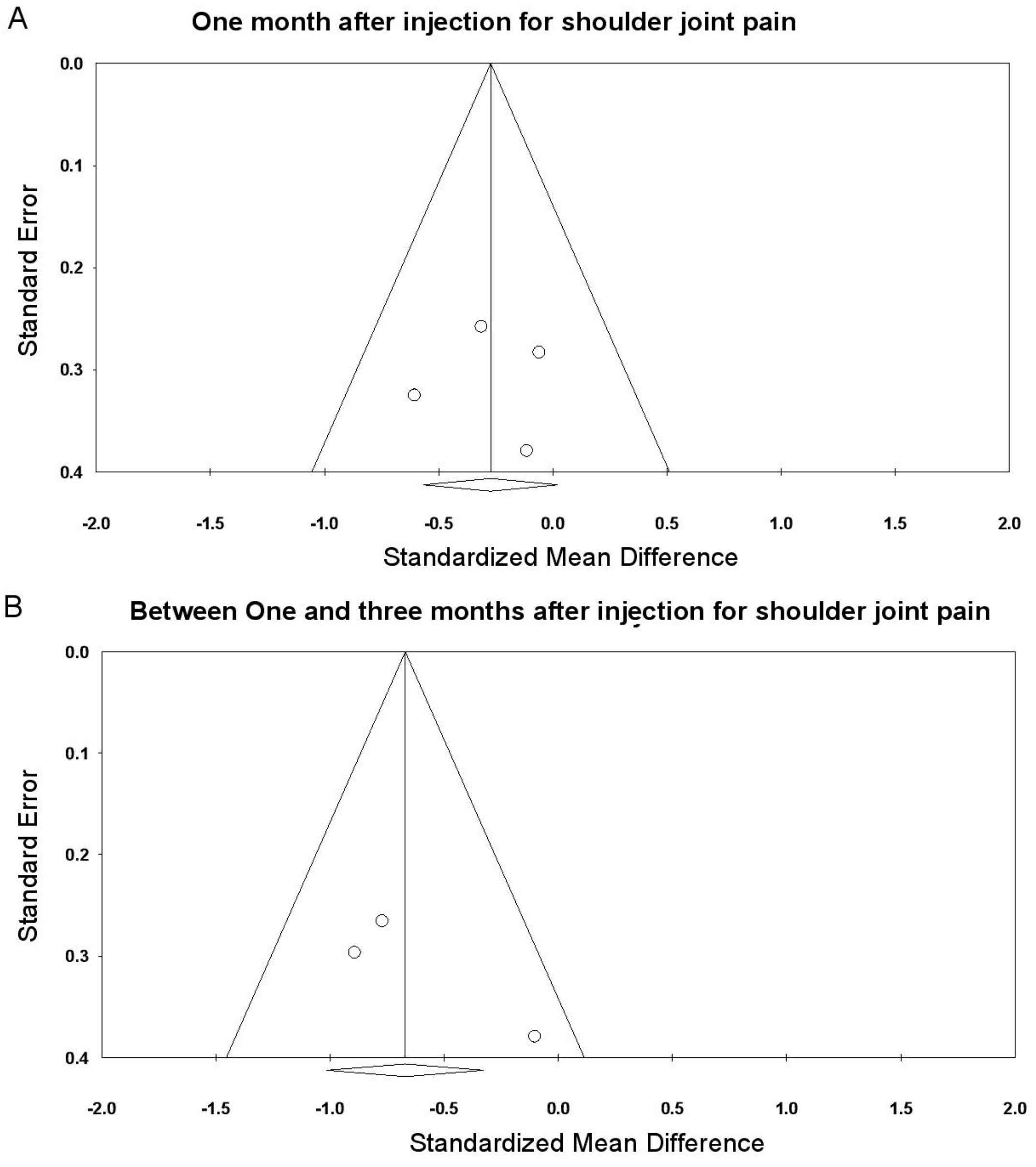

Figure S1. Funnel plot for the comparisons of the standardized mean difference at (**A**) one month and (**B**) between one and three months after injection for shoulder joint pain.

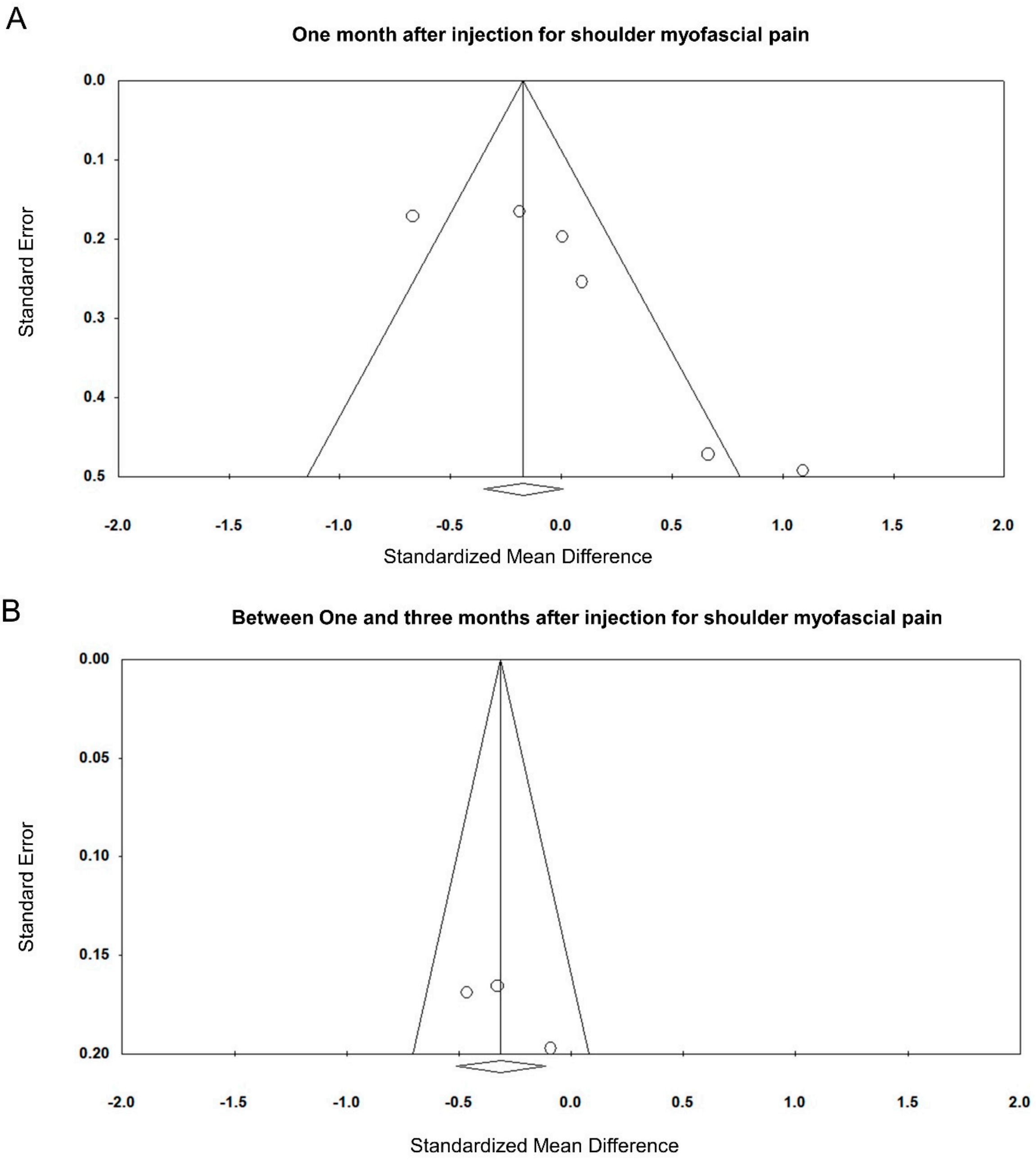

Figure S2. Funnel plot for the comparisons of the standardized mean difference at (**A**) one month and (**B**) between one and three months after injection for shoulder myofascial pain.
